# Protocol for imbibed seed piercing for *Agrobacterium*-mediated transformation of jute

**DOI:** 10.1016/j.xpro.2023.102767

**Published:** 2023-12-11

**Authors:** Shuvobrata Majumder, Subhadarshini Parida, Nrisingha Dey

**Affiliations:** 1 Institute of Life Sciences, Nalco square, Bhubaneswar, Odisha 751023, India

**Keywords:** Genetics, Model Organisms, Plant sciences, Molecular Biology, Gene Expression, Biotechnology and bioengineering

## Abstract

Here, we present a streamlined *Agrobacterium*-mediated transformation protocol for jute (*Corchorus* sp.). We describe steps to pierce and vacuum infiltrate imbibed jute seeds with *Agrobacterium* suspension. We then detail procedures for selecting transformed seeds by using a hygromycin-B-supplemented medium. This approach can achieve transformation efficiencies of 20.44% ± 1.17% and 15.55% ± 0.58% for tossa (*C. olitorius*) and white (*C. capsularis*) jute, respectively. Demanding minimal resources and time, this protocol can elevate genetic engineering research in jute fiber crops.

For complete details on the use and execution of this protocol, please refer to Majumder et al. (2020).^1^

## Highlights

• Protocol for imbibed jute seeds piercing for *Agrobacterium*-mediated transformation
• Detailed steps on co-cultivation and infiltration of *Agrobacterium*
• Steps to select and validate jute transformation

## Abstract (editor highlights)

Publisher’s note: Undertaking any experimental protocol requires adherence to local institutional guidelines for laboratory safety and ethics.

## Graphical abstract

**Figure:**
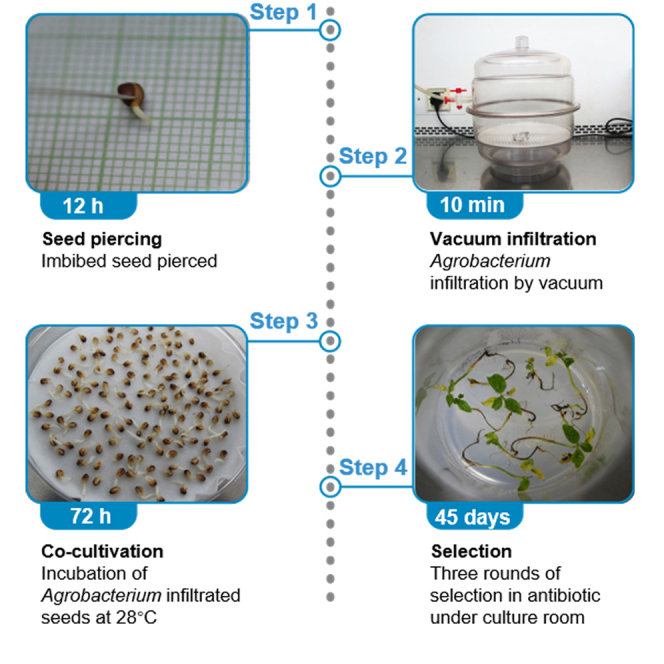

## Before you begin

Jute holds immense potential as a global bast fiber crop. However, limited genetic engineering research presents bottleneck conditions in jute improvement for diversified applications. This opens exciting avenues for them who have an easy, efficient, reliable, and reproducible transformation protocol. *Agrobacterium*-mediated transformation (AMT) protocols are more commonly used in jute than any other method of plant transformation protocols.^2^ Currently, the shoot tip (explant) method of *Agrobacterium*-mediated transformation in jute is used for transgenic jute development.^3^^,^^4^^,^^5^^,^^6^^,^^7^ Time duration, tissue culture dependency for regeneration of the shoot tip to a plant, and transformation efficiency are the limitations of the current shoot tip method, despite it being a stable and efficient method. Overcoming these limitations, here we present an improved plant genetic transformation protocol for jute that is easy, reliable, reproducible, less time-consuming in explant preparation, with fewer media and resource requirements, partial dependency on tissue culture, and facilitating a higher transformation efficiency. This protocol is known as the imbibed seed piercing method (ISPM) for AMT in jute and offers a foundation for stable and successful jute crop improvement, fulfilling the high demand for strong, long, and environment friendly fibers.

Overall, in the ISPM for AMT protocol, jute seeds are subjected to surface sterilization, followed by overnight imbibition in water and piercing with a needle to facilitate the penetration of *Agrobacterium* cell suspension through vacuum infiltration. Transformed jute seeds are selected by growing them in a medium supplemented with hygromycin-B. The transgene integration is confirmed through PCR analysis. PCR-based plant genotyping minimizes the risk of selecting false-positive plants during the antibiotic-based selection of transformants. The combined use of three consecutive rounds of selection in a hygromycin-B antibiotic-supplemented medium and PCR screening provides a reliable method to validate the actual transformation efficiency of the ISPM for AMT protocol in jute. A similar approach (combination of hygromycin-B and PCR screening) is popularly applied for the successful and rapid selection of other (homozygous) transgenic plants.^8^

The ISPM for AMT is showing successfully results on white jute (*C. capsularis*) cultivar JRC321, tossa jute (*C. olitorius*) cultivar JRO204, and flax (*Linum usitatissimum*) cultivar FT-897, with gene transformation efficiencies of 10.22%,^1^ 9.60%,^9^ and 7.33%,^1^ respectively. This protocol encompasses the ISPM for AMT, tested on tossa jute cultivar JRO524 and white jute cultivar JRC212.

### Check seed germination percentage

**Timing: 2–4 days**

1. Before using jute seeds for sample preparation, check their germination percentage by growing them in moist filter paper in an incubation of 24 h in 37°C.

***Note:*** Freshly harvested jute seeds are preferable for the transformation.

***Note:*** If the germination percentage is more than 90% then the seeds should be used for the experiment.

***Alternatives:*** Check the germination efficiency by sowing the seeds in soil. In this case it will take 3–4 days to check germination percentage after the seedlings develop.

### Transformation of *Agrobacterium* with vector of interest

**Timing: 2 days**

2. Use the freeze/thaw method^10^ to introduce the plant transformation vector, pCAMBIA1301 (Abcam, UK, Cat# ab275753), into the *Agrobacterium tumefaciens* strain LBA4404 competent cells (Takara, Japan, Cat# 9115).

## Key resources table

**Table:** 

REAGENT or RESOURCE	SOURCE	IDENTIFIER
**Bacterial and virus strains**		

*Agrobacterium tumefaciens* strain LBA4404	Takara, Japan	Cat# 9115

**Chemicals, peptides, and recombinant proteins**		

Acetosyringone	HiMedia Laboratories, India	Cat# PCT1301-5G
Agar powder	HiMedia Laboratories, India	Cat# PCT0901-1KG
Agarose	Promega, USA	Cat# V315
Bavistin (carbendazim 50%) powder	Crystal, India	CIR-14
Dimethyl sulfoxide (DMSO)	HiMedia Laboratories, India	Cat# TC185-250ML
DNA ladder (1 kb)	Thermo Scientific, USA	Cat# SM0311
Ethanol	Supelco, USA	Cat# 1009831000
Ethidium bromide	HiMedia Laboratories, India	Cat# MB071-5G
Hygromycin-B solution (50 mg/mL stock)	HiMedia Laboratories, India	Cat# PCT1503-20ML
Kanamycin antibiotic	HiMedia Laboratories, India	Cat# A009-20ML
Luria broth (LB) media	HiMedia Laboratories, India	Cat# M575-500G
Methanol	Supelco, USA	Cat# 1060091000
Murashige and Skoog basal medium and vitamins	Sigma-Aldrich, USA	Cat# M5519-50L
Myo-inositol	Sigma-Aldrich, USA	Cat# I7508-100G
Nuclease-free water	Promega, USA	Cat# P119E
Rifampicin	HiMedia Laboratories, India	Cat# PCT1119-100MG
Sodium hypochlorite (4%)	HiMedia Laboratories, India	Cat# PCT1311
Sucrose	HiMedia Laboratories, India	Cat# PCT0607-1KG
TAE buffer (50×)	Thermo Scientific, USA	Cat# B49
Timentin antibiotic	HiMedia Laboratories, India	Cat# PCT1113-2G
Tween 20	HiMedia Laboratories, India	Cat# MB067

**Critical commercial assays**		

PCR master mix	Takara, Japan	Cat# RR310
Plant DNA isolation kit	Macherey-Nagel (MN), Germany	Cat# 740770.50

**Experimental models: Organisms/strains**		

Seeds of *C. capsularis*	Central Research Institute for Jute and Allied Fibres (CRIJAF), India	JRC212
Seeds of *C. olitorius*	CRIJAF, India	JRO524

**Oligonucleotides**		

Forward primer (5′-3′) GAAATTGCCGTCAACCAAGCTC	IDT, USA	In this study
Reverse primer (5′-3′) GCGAGAGCCTGACCTATTGCATC	IDT, USA	In this study

**Recombinant DNA**		

Plant transformation vector pCAMBIA1301	Abcam, UK	Cat# ab275753

**Software and algorithms**		

Microsoft Excel Worksheet	Microsoft, USA	N/A
PrimerQuest Tool	IDT, USA	N/A

**Other**		

Aluminum foil	Hindalco, India	FRESHWRNEW01
Autoclave	Jeio Tech, Korea	ST-65G
Beaker 100 mL	Borosil, India	Cat# 1000029
Benchtop centrifuge for 0.2 mL (PCR) tubes	Heathrow Scientific, USA	Sprout
Benchtop centrifuge for 2.0 mL tubes	Thermo Scientific, USA	Heraeus Fresco 17
Centrifuge tube 0.2 mL	Tarsons, India	Cat# 510051
Centrifuge tube 1.5 mL	Axygen, USA	Cat# MCT-150-C
Centrifuge tube 50 mL	Tarsons, India	Cat# 546041
Conical flask 100 mL	Borosil, India	Cat# 4980016
Conical flask 100 mL (amber)	Borosil, India	Cat# 4989016
Conical flask 250 mL	Borosil, India	Cat# 4980021
Cooled tabletop centrifuge for 50 mL tubes	Eppendorf, Germany	5810R
Culture room	ILS, India facility	N/A
Electrophoresis system	Bio-Rad, USA	PowerPac Basic
Gel documentation system	Bio-Rad, USA	ChemiDoc MP
Gloves	Kimberly-Clark, Canada	Cat# 55083
Greenhouse	ILS, India facility	N/A
Hotplate	Neuation, India	iSTIR HP550 Prime
Incubator shaker	BEING Scientific, USA	THZ-984
Laminar air flow	Thermo Scientific, USA	MSC 1.2
Magenta box (square)	HiMedia Laboratories, India	Cat# PW1138-50NO
Magnetic stirrer	Genei, India	LI-HP-MS-200
Measuring cylinder 10 mL	Borosil, India	Cat# 3021006
Measuring cylinder 500 mL	Borosil, India	Cat# 3021024
Microtips 10 μL	ABclonal, USA	Cat# AI10CP
Microtips 1000 μL	ABclonal, USA	Cat# AI1000CP
Microtips 200 μL	ABclonal, USA	Cat# AI200CP
Microwave appliance	LG Electronics	MC-808WAR
Mortar and pestle	Local vendor (India)	N/A
NanoDrop	Thermo Scientific, USA	NanoDrop 2000C
Non-absorbent cotton roll (used for cotton plug preparation)	EQWell, India	B0BRPZR49W (Amazon.in)
Parafilm	Amcor, Australia	Cat# PM-996
PCR thermal cycler	Applied Biosystems, USA	Proflex PCR System
Petri plate (100 mm)	Corning, USA	Cat# BP94A-01
Petri plate (35 mm)	Tarsons, India	Cat# 460035-35MM
pH meter	Oakton, India	pH 700 Benchtop
Phyta jar (round)	HiMedia Laboratories, India	Cat# PW1125-50NO
Pipette set	Eppendorf, Germany	Research Plus
Pump for desiccator	Tarsons, India	Rockyvac 410
Reagent bottle 100 mL	Borosil, India	Cat# 1501016
Reagent bottle 1000 mL	Borosil, India	Cat# 1501029
Reagent bottle 50 mL	Borosil, India	Cat# 1501012
Reagent bottle 500 mL	Borosil, India	Cat# 1501024
Soilrite-mix	Keltech Energies, India	B08M3QZYFN (Amazon.in)
Spectrophotometer (bacterial OD)	Eppendorf, Germany	Biophotometer Plus
Stainless forceps	HiMedia Laboratories, India	Cat# LA706
Stainless sewing needle	PMW, India	UK-BZJS-0112-121 (Amazon.in)
Syringe	Dispo Van, India	5 mL (Disposable)
Syringe filter (0.22 μm)	Millipore, USA	Cat# SLGP033RS
Tube (bacteriology) 55 mL	Borosil, India	Cat# 9820U08
Vacuum desiccator (diameter 250 mm)	Tarsons, India	Cat# 402030
Water purification system (for ddH_2_O/Milli-Q water)	Millipore, USA	Elix
Weight balance	Sartorius, Germany	CP423S
Whatman No. 1 filter paper	GE Healthcare, USA	Cat# 1001-090

## Materials and equipment

### Short recipes

**Kanamycin antibiotic stock solution (50 mg/mL):**

• Dissolve 50 mg of kanamycin powder (HiMedia Lab., India Cat# A009-20ML) in 1 mL of ddH_2_O (Milli-Q water) and filter-sterilize it (Millipore, Cat# SLGP033RS).

***Note:*** The final molar concentration (M) of the kanamycin stock is 0.0857 M.

**CRITICAL:** Use kanamycin antibiotic to select the pCAMBIA1301 vector.

**Pause point:** Store this stock solution at 4°C and use it within 1 month.

**Rifampicin antibiotic stock solution (25 mg/mL):**

• Dissolve 25 mg of rifampicin powder (HiMedia Lab., India Cat# PCT1119-100MG) in 1 mL of methanol (Supelco Cat# 1060091000) and filter-sterilize it (Millipore, Cat# SLGP033RS).

***Note:*** The final molar concentration (M) of the rifampicin stock is 0.0304 M.

**CRITICAL:** Use rifampicin antibiotic to select the *Agrobacterium tumefaciens* strain LBA4404.

**Pause point:** Store this stock solution at 4°C and use it within 1 month.

**Acetosyringone stock solution (100 mM stock):**

• Dissolve 192 mg of acetosyringone powder in 10 mL of DMSO and filter-sterilize it (Millipore, Cat# SLGP033RS).

**Pause point:** Store this stock solution at 4°C and use it within 3 months.

**Timentin stock solution (500 mg/mL stock):**

• Dissolve 500 mg of timentin powder in 1 mL of Milli-Q water and filter-sterilize it (Millipore, Cat# SLGP033RS).

***Note:*** The final molar concentration (M) of the timentin stock is 0.857 M.

**CRITICAL:** Use timentin antibiotic to remove bacterial contamination, including *Agrobacterium tumefaciens* strain LBA4404.

**Pause point:** Store this stock solution at 4°C and use it within 72 h.

**Culture media for *Agrobacterium*:**

**CRITICAL:** The culture of *Agrobacterium tumefaciens* strain LBA4404, which harbors the pCAMBIA1301 plant transformation vector, is made using Luria broth (HiMedia Lab., India, Cat# M575-500G) supplemented with kanamycin and rifampicin antibiotics. The pCAMBIA1301 vector carries the kanamycin resistance gene, while the *Agrobacterium* LBA4404 strain possesses the rifampicin resistance gene.

• Using the following components, prepare culture media to grow *Agrobacterium* cells.

**Table:** 

Reagent	Final concentration (per liter)	Amount (per 100 mL)
Luria broth (LB) powder	20 g/L	2 g
Kanamycin (50 mg/mL stock)	50 mg/L	100 μL
Rifampicin (25 mg/mL stock)	25 mg/L	100 μL
ddH_2_O (Milli-Q water)	N/A	100 mL
**Total Volume**	N/A	100 mL

**CRITICAL:** After autoclaving, add kanamycin and rifampicin antibiotics (previously filter sterilized) to the medium, only after it has reached approximately temperature around 45°C.

**Pause point:** Store this medium at 4°C and use within 2 weeks.

### Infiltration & co-cultivation media

**CRITICAL:** This liquid medium facilitates the infiltration of *Agrobacterium* cells into the seeds and supports their growth during the co-cultivation period. Acetosyringone is an important part of the medium that helps *vir* genes become active in *Agrobacterium* cells, which speeds up the transformation process.^11^ The absence of acetosyringone in the medium leads to necrosis and browning of the explant.^3^

• Using the following components, prepare the ‘Infiltration & Co-cultivation**’** medium.

**Table:** 

Reagent	Final concentration (per liter)	Amount (per liter)
MS salts and vitamins	Full strength	4.4 g
Myo-inositol	0.1%	1 g
Acetosyringone (100 mM stock)	100 μM	100 μL
Sucrose	2%	20 g
ddH_2_O (Milli-Q water)	N/A	1000 mL
**Total Volume**	N/A	1000 mL

• Adjust pH to 5.6 with 2 M NaOH solution.
• Autoclave the medium at 121°C for 20 min at 15 lbs pressure.

***Note:*** Add acetosyringone (filter sterilized) to the medium after autoclaving and just before use, only when it reaches temperature around 45°C.

**Pause point:** Store the medium at 4°C and use within 2 weeks.

### Agro-washing media

**CRITICAL:** This medium eliminates *Agrobacterium* cells after the co-cultivation step in the transformation process. The precise concentration of the timentin antibiotic is key to ensuring the complete removal of *Agrobacterium* cells, preventing their undesirable overgrowth, which can hinder the subsequent steps.

• Using the following components, prepare the ‘Agro-washing**’** medium.

**Table:** 

Reagent	Final concentration (per liter)	Amount (per liter)
MS salts and vitamins	Half strength	2.2 g
Sucrose	1%	10 g
Timentin antibiotic (500 mg/mL Stock)	500 mg/L	1 mL
ddH_2_O (Milli-Q water)	N/A	999 mL
**Total Volume**	N/A	1000 mL

***Note:*** Adjust the pH to 5.6 with 2 M NaOH solution before you autoclave.

***Note:*** Add timentin antibiotic (filter sterilized) to the autoclaved media after it reaches temperature around 45°C.

**Pause point:** Store the medium at 4°C and use within 2 weeks.

### Selection media

**CRITICAL:** The purpose of this hygromycin-B antibiotic-supplemented medium is to select transgenic jute plants. The transformation vector pCAMBIA1301 carries the hygromycin-B resistance gene (*hptII*), which is successfully transferred to transgenic jute plants during transformation. These transgenic plants can survive and grow in this medium. Notably, tossa jute (JRO524) selection requires a higher hygromycin-B concentration compared to white jute (JRC212).

• Using the following components, prepare the selection medium.

**Table:** 

Reagent	Final concentration (per liter)	Amount (per liter)
MS salts and vitamins	Full strength	4.4 g
Sucrose	1.5%	15 g
Hygromycin-B antibiotic (50 mg/mL stock)	15 mg/L (for JRC212) 18 mg/L (for JRO524)	300 μL (for JRC212) 360 μL (for JRO524)
Timentin antibiotic (500 mg/L Stock)	250 mg/L	500 μL
Agar powder	0.8%	8 g
ddH_2_O (Milli-Q water)	N/A	1000 mL
**Total Volume**	N/A	1000 mL

***Note:*** Adjust pH to 5.8 with 2 M NaOH solution before autoclave.

***Note:*** Add hygromycin-B and timentin antibiotics to the autoclaved media after it reaches temperature around 45°C.

**Pause point:** Store the medium at 4°C and use within 2 weeks.

***Note:*** The same medium is used as rooting medium without the hygromycin-B and timentin antibiotics.

### Agarose gel preparation

• Mix the following components to prepare the agarose gel.

**Table:** 

Reagent	Final concentration	Amount (per 100 mL)
Agarose powder	1%	1 g
TAE Buffer (50× stock)	1×	2 mL
ddH_2_O (Milli-Q water)	N/A	98 mL
**Total Volume**	N/A	100 mL

• Dissolve the components by heating (75°C–80°C) using a hotplate or microwave.
• When solution temperature is around 45°C, add 2 μL of ethidium bromide (from the stock of 100 mg/mL).
• Keep aside at 22°C–25°C temperature for solidification.

***Note:*** For electrophoresis buffers use 1× TAE (Thermo Scientific, USA Cat# B49).

## Step-by-step method details

Most of the steps require maintaining sterile conditions and using a laminar workbench. Any steps that can be performed outside of the laminar workbench are specifically mentioned.

### Seed surface sterilization of jute

**Timing: 1.5–2 h**

This step describes how to remove contamination from the jute seeds. Fungal and bacterial contamination of the seeds needs to be controlled at the very beginning of the experiment without damaging the jute seeds. This step describes the protocol of jute sterilization by the application of Bavistin (carbendazim 50%) fungicides, ethanol, sodium hypochlorite and Tween-20.

1. Examine JRC212 and JRO524 jute seeds to select only those that are filled and healthy.
2. Immerse 150 jute seeds in 5 mL of 0.1% Bavistin solution, a commercially available fungicide, for 10 min.

**CRITICAL:** For steps 3–8, perform the procedures within a laminar workbench to maintain aseptic conditions. Proper care should be taken to avoid any contamination during the process.

3. Remove seeds from the Bavistin solution.
4. Wash the selected seeds twice with sterile water for 2 min in a 100 mL conical flask.
5. Immerse seeds in 70% ethanol for 4 min with continuous stirring.
6. Wash seeds with sterile water for 2 min to remove residual ethanol.
7. Prepare a mixture (50 mL) of 2% sodium hypochlorite with 2–3 drops of Tween-20.
  a. Immerse seeds in this solution for 15 min with continuous stirring.
  b. Cover the conical flask with aluminum foil or use an amber-colored conical flask to avoid light exposure.

***Note:*** Seal the flask's mouth with Parafilm to prevent contamination when stirring using a magnetic stirrer outside of the laminar.

***Alternatives:*** Instead of a magnetic stirrer, a shaker incubator can also be used, but with a magnetic stirrer, better results are observed.

**CRITICAL:** Adjust the stirring speed to prevent excessive seed coat detachment from the jute seeds (Methods video S1).

Methods video S1. Surface sterilization of jute seeds—Demonstrates the surface sterilization process for jute seeds in preparation for *Agrobacterium*-mediated transformation, related to step 7

***Note:*** The change in solution color from pale yellow to light brown is due to the leaching of seed coat color in the solution.

8. Wash seeds 3–4 times with sterile water for 5 min each to thoroughly rinse off the sodium hypochlorite solution.

***Note:*** Continue rinsing thoroughly such that no more foaming (developed by Tween-20 remains) is visible and ensuring that no foam is produced during the final wash, also, getting rid of any residual smell of sodium hypochlorite solution.

9. Place the sterilized seeds on sterile Whatman No. 1 filter paper (GE Healthcare, USA Cat# 1001-090) to remove excess water.

***Note:*** Remove any damaged seeds or any seeds with detached cotyledons in this step.

### Jute seed germination

**Timing: 12–24 h**

This step describes explant preparation for the *Agrobacterium*-mediated jute transformation. Here the explant is germinated jute seeds. Only water is required as germination medium for the jute seeds.

**CRITICAL:** Perform the following steps in a laminar workbench to maintain aseptic conditions.

10. Place the sterilized jute seeds on a filter paper within a petri plate (100 mm).
11. Add 3 mL of sterile water to the petri plate containing the seeds.
  a. Seal the petri plate with Parafilm.
  b. Cover it with aluminum foil to prevent exposure to light.

***Note:*** The amount of water required for germination depends on the quantity and size of seeds used in the experiment. For 100–150 seeds, 3 mL is sufficient, and it should be increased as the number of jute seeds increases.

12. Put the petri plate in an incubator (Being Scientific, USA THZ-98A) at 37°C for a duration of 12–24 h.

***Note:*** The duration of incubation may vary based on the specific seed cultivars. Tossa jute (*C. olitorius*) typically requires more time compared to white jute (*C. capsularis*) cultivars.

***Alternatives:*** Incubation can also be carried out at 22°C–25°C temperature. However, in such cases, a slightly longer incubation period might be necessary.

### Preparation of bacterial culture media

**Timing: 2 h**

This step describes the process of culture media preparation for *Agrobacterium tumefaciens.* The Luria broth (LB) powder is weighed, dissolved in water and divided into glass tubes. The tubes are sealed and autoclaved. Later kanamycin is added to the media before it solidifies. At the end of this step, we will receive a medium where the *Agrobacterium* can be selectively grown.

13. Weigh 2.0 g of LB powder (HiMedia, India).
14. Dissolve it in 100 mL of distilled water.
15. Distribute 10 mL of the media into each glass tube (Borosil, India Cat# 9820U08).
16. Securely seal the tubes with cotton plugs.
17. Autoclave the tubes at 121°C for 20 min at 15 lbs pressure (using a Jeio Tech ST-65G).
18. After autoclaving, add 10 μL of kanamycin (HiMedia, India) to the 10 mL LB media.

***Note:*** Add the kanamycin antibiotic to the autoclaved media after it reaches around 45°C.

**Pause point:** Store the autoclaved LB media-containing tubes at 4°C and use them within 2 weeks.

### Culture of *Agrobacterium* LBA4404 cells harboring pCAMBIA1301 plant transformation vector

**Timing: 8–12 h**

This step highlights the process of *Agrobacterium tumefaciens* LBA4404 culture in the above-mentioned LB media. Kanamycin containing LB media is used to grow *Agrobacterium* cells harboring pCAMBIA1301 plasmid which contain the kanamycin resistant maker gene (*nptII*).

19. Add 10 μL of *Agrobacterium* LBA4404 primary culture to 10 mL of LB media containing kanamycin.
20. Incubate the mixture in an incubator shaker at 28°C for 12 h.
21. Measure the optical density (OD) of *Agrobacterium* LBA4404 culture at 600 nm using a spectrophotometer (Eppendorf, Germany, Model: Biophotometer Plus).
22. Transfer the culture to a sterile 50 mL centrifuge falcon tube (Tarsons, India Cat# 546041).
23. Centrifuge it at 2600 × *g* for 5 min at 10°C in a centrifuge (Eppendorf, Germany, Model# 5810R) to pellet the *Agrobacterium* cells.
24. Resuspend the pellet in fresh LB media to achieve an OD 0.3; this is equivalent to approximately 1.5 × 10^8^ cells/mL.

***Alternatives:*** A full loop of *Agrobacterium* culture from a 4°C stored culture plate can be directly resuspended in fresh ‘infiltration and co-cultivation medium’ to achieve OD 0.3. In this case, there is no need for a 12–14 h grown culture, which is a time saver.

### The imbibed seed piercing method (ISPM) of *Agrobacterium*-mediated transformation (AMT)

**Timing: 2 months**

This major step describes *Agrobacterium*-mediated transformation in jute. Germinated jute seeds are used as explant. A tiny hole is made by piercing the seeds. This facilitates penetration of *Agrobacterium* cells into the seeds during the vacuum infiltration process. At the end of this step T_0_ plants will be developed and grow in the greenhouse.

**CRITICAL:** Perform the following step 25 to 33, step 35 to 42, and step 44 to 45 in a laminar air flow work bench to maintain aseptic conditions

25. Pierce imbibed jute seeds with a sterilized needle near the apical meristematic zone of the plumule (Methods video S2).
  Methods video S2. Piercing of imbibed jute seeds—Illustrates the technique of piercing imbibed jute seeds, a crucial step in the transformation protocol, related to step 25

**CRITICAL:** Needles must be flame sterilized and allowed to cool periodically before each piercing. Each seed can be pierced a maximum of two times without permanent damage.

26. Soak the pierced seeds in the 'infiltration and co-cultivation medium'.
27. Cover the soaked seeds with aluminum foil and incubate for 30 min to 1 h in a laminar workbench at 22°C–25°C temperature.

***Note:*** The 'infiltration and co-cultivation medium' contains *Agrobacterium* cells (OD 0.3), therefore gentle agitation is necessary during incubation to ensure the cells remain suspended and make contact with the pierced seeds.

***Note:*** Acetosyringone should be added to the 'infiltration and co-cultivation medium' before introducing the pierced seeds. To ensure proper mixing, pipette the medium 10–20 times.

***Note:*** The volume of media required depends on the number of seeds used. Typically, 25 mL is suitable for a 100 mm petri plate (Corning, USA Cat# BP94A-01), while 6 mL is sufficient for a 35 mm plate (Tarsons, India Cat# 460035-35MM).

28. Put the petri plates, which contain pierced seeds submerged in medium, into a vacuum desiccator chamber, and vacuum infiltrate for 10 min at a pressure of 550 mm Hg (Methods video S3).
  Methods video S3. Vacuum infiltration method of *Agrobacterium* transformation—Provides a visual guide to the vacuum infiltration method used for introducing Agrobacterium into pierced jute seeds during the transformation process, related to step 28

***Note:*** Ensure that the desiccator is properly cleansed with 70% ethanol and exposed to UV light for 30 min under laminar airflow before use.

**CRITICAL:** Vacuum infiltration should not exceed 20 min, to avoid the risk of tissue damage.

29. Place the inoculated seeds on sterile Whatman No. 1 filter paper to remove excess *Agrobacterium* infiltrate.

***Alternatives:*** Sterile blotting paper can also be used in place of filter paper

30. Incubate for 5 min to achieve a semi-dry state (until the excess liquid medium is removed from the seed surface).
31. Transfer seeds to a 100 mm petri plate on Whatman No. 1 filter paper.
32. Saturate the filter paper with 3 mL of co-cultivation medium.
33. Seal the petri plate with Parafilm and cover with aluminum foil.
34. Incubate at 28°C in the dark for 72 h for co-cultivation.

***Note:*** The co-cultivation medium here is the same as that in step 26, but without *Agrobacterium* cells.

**CRITICAL:** Ensure that the amount of media is neither excessive nor scarce. Excess media can lead to seed browning and death, while a shortage of media can cause seeds to dry out and die.

35. After co-cultivation, transfer the seeds to a fresh 100 mm petri plate.
36. Wash the seeds four times for 5 min each, using only 'Agro-washing medium' without any antibiotic.

***Note:*** While sterile water can be used for this washing step, using media is recommended as it yields better results.

37. Submerge the seeds in 'Agro-washing medium' with timentin antibiotic (HiMedia Lab., India Cat# PCT1113-2G).

***Alternatives:*** 1 g/L cefotaxime (HiMedia Lab., India Cat# MB134) can be used as an alternative to timentin for this step.

38. Incubate the seeds for 20 min.
39. Vacuum infiltrate the seeds for 5 min at a pressure of 550 mm Hg.
40. Carefully remove the seeds from the antibiotic-containing medium and place them on sterile Whatman No. 1 filter paper.
41. Incubate the seeds for 45 min to ensure proper drying.
42. Transfer the seeds into round screw-cap phyta jars (HiMedia Lab., India Cat# PW1125) containing hygromycin-B (HiMedia Lab., India Cat# PCT1503) in the 'selection medium'.
43. Cultivate them for a duration of 14 days under controlled culture room conditions: a temperature of 27 ± 2°C, a photoperiod of 16 h of light, and 8 h of darkness.

***Alternatives:*** Cultivation can be done in petri plates, but it is preferable to use transparent screw-cap bottles or phyta jars for this step, as plants may touch the lids and become bent in the petri plates.

**CRITICAL:** The sub-lethal selection concentration of hygromycin-B may vary depending on the cultivar used and should be optimized accordingly. Use 15 mg/L hygromycin-B for JRC212 seeds, and 18 mg/L hygromycin-B for JRO524 seeds.

***Note:*** The selection media should be freshly prepared, and hygromycin-B should be added after the autoclaved media cools down (around 45°C) but not solidifies.

44. Transfer the surviving seedlings once more to the freshly prepared 'selection media' for an additional 14 days as a second round of selection.

***Note:*** These plants should continue to grow under the same conditions as step 43.

**CRITICAL:** It is recommended to transfer five or six plants to each magenta box (HiMedia Lab., India Cat# PW1138) during this step. This ensures that the plants have sufficient space and nutrients for optimal growth.

45. Transfer the surviving seedlings once again, this time for a third round of selection in media containing hygromycin under the same conditions as step 43 for another 14 days.

***Note:*** Performing three rounds of selection significantly increases the chances of selecting the most transformed plants. However, if time is a concern, minimize the duration of the selection process by performing only up to the second round. Nevertheless, it is highly recommended to conduct three rounds of selection, each lasting 12–14 days, as hygromycin-B effectiveness diminishes over longer periods, increasing the risk of selecting false-positive plants.

46. Allow the plants to grow for 10 days in a medium that does not contain hygromycin-B.

***Note:*** This step is primarily to encourage root development.

***Alternatives:*** This step is optional and can be skipped if the plants have already developed a healthy root system during the selection process.

47. Clean the roots of the plants thoroughly using sterile water.
48. Plant them in soil in the greenhouse to facilitate further growth and maturation.

**CRITICAL:** Take care when cleaning the roots to avoid any damage. Transferring plants to soil with media stuck to roots, increases the risk of fungal contamination.

### Jute genomic DNA isolation

**Timing: 2 h**

This step highlights the process of genomic DNA isolation from greenhouse grown transgenic and control jute plants. This is a kit-based plant DNA isolation method but as alternative method of jute DNA isolation the Miniprep^12^ method can also be used.

49. Collect fully opened young leaves from each plant for genomic DNA isolation.
50. Wash the leaves twice with ddH_2_O and blot dry.
51. Use a commercial plant DNA isolation kit (Macherey-Nagel, Germany Cat# 740770) and isolate genomic DNA by following the kit’s instructions.

***Note:*** The kit’s instructions (DNA isolation protocol) are available at the link.

52. Measure the concentration of isolated DNA using a NanoDrop spectrophotometer (Thermo Scientific, USA NanoDrop 2000C).

### PCR primer design

**Timing: 30 min**

This step highlights PCR primer design for *hptII* gene (hygromycin phosphotransferase) of pCAMBIA1301 using the online tool-PrimerQuest (free access). The accession number for the gene sequence to be downloaded is provided below. After this step the primer sequences are generated.

53. Download the sequence of the hygromycin-B-resistant *hptII* gene of pCAMBIA1301 using GenBank: AF234297 (Data S1).
54. Use the *hptII* gene sequence as the input for primer design in the PrimerQuest Tool, available at the link.
55. Run the tool using its default settings for the PCR primers.

***Alternatives:*** Primer3web (https://primer3.ut.ee) is another (free access) software for primer designing.

56. Select a primer set from the list of multiple suggestions provided in the PrimerQuest Tool.
57. Order the forward (5′-GAAATTGCCGTCAACCAAGCTC-3′) and reverse (5′-GCGAGAGCCTGACCTATTGCATC-3′) primers for synthesis from IDT, USA.

***Alternatives:*** Primers can be ordered from any reliable service provider.

### Screening of *hptII* gene in transformed jute plants though PCR

**Timing: 3 h**

This step describes the PCR conditions for the process of amplification of the *hptII* gene. This is a validation step for the transgenic integration of the *hptII* gene in the jute genome.

58. In a sterile 1.5 mL centrifuge tube, mix the following reagents. Thaw reagents on ice and keep reagents on ice until use.

**Table:** PCR reaction master mix

Reagent	Final concentration	Amount (per 25 μL reaction)
PCR Master mix (2×)	1×	12.5 μL
Forward primer (GAAATTGCCGTCAACCAAGCTC)	0.2 μM	1 μL
Reverse primer (GCGAGAGCCTGACCTATTGCATC)	0.2 μM	1 μL
Plant genomic DNA	100 ng/μL	1 μL
Nuclease-free water	N/A	9.5 μL
**Total Volume**	N/A	25 μL

***Note:*** Prepare a working stock of 100 ng/μL of plant genomic DNA to use as a sample in the PCR reaction mixture.

***Note:*** Mix all the components by repetitive pipetting and give a pulse spin before putting into the thermal cycler.

59. Distribute 24 μL of PCR master mix into 0.2 mL PCR tubes.
60. In each PCR tube, add and mix by pipetting 1 μL of genomic DNA (100 ng/μL) from the tested plant.
61. Set the program as follows in a thermal cycler (Applied Biosystems, USA Proflex PCR System) for 35 cycles.

**Table:** PCR cycling conditions

Steps	Temperature	Time	Cycles
Initial Denaturation	98°C	10 s	1
Denaturation	98°C	10 s	35 cycles
Annealing	62.6°C	30 s	
Extension	72°C	30 s	
Final extension	72°C	5 min	1
Hold	4°C	Forever

62. Run the PCR products on a 1% agarose gel using an electrophoresis system (Bio-Rad, USA Model: PowerPac Basic).
63. Determine the band size by comparing it with a 1 kb DNA ladder (Thermo Scientific, USA Cat# SM0311).
64. In the agarose gel, a 599 bp amplified product of the *hptII* gene should be visible.

## Expected outcomes

**Figure 1:**
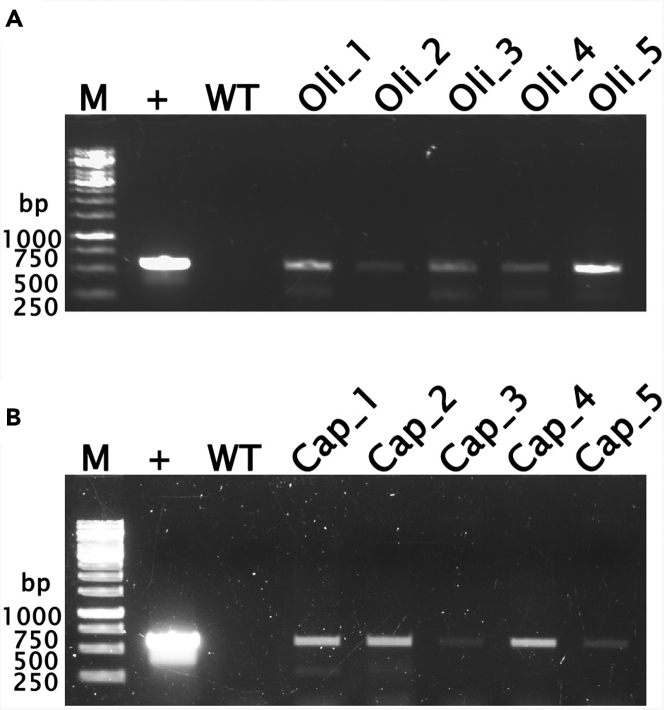
PCR confirmation of *hptII* gene integration in transformed jute cultivars (A) PCR results for tossa jute cultivar JRO524 and (B) white jute cultivar JRC212. In both panels (A) and (B), 'M' represents the 1 Kb DNA ladder (Thermo Scientific, USA Cat# SM0311). The '+' symbol designates the positive control- pCAMBIA1301 vector plasmid (Abcam, UK Cat# ab275753). In the other lanes, '*hptII* positive' indicates 599 bp PCR products from transformed plants of *Corchorus olitorius* (Oli) and *Corchorus capsularis* (Cap).

After three rounds of selection with the hygromycin-B antibiotic, the surviving plants should be healthy and test PCR-positive for the hygromycin-B-resistant *hptII* gene. This is confirmed by the visualization of a 599-bp amplified product of the *hptII* gene in agarose gel (Figure 1).

The mean transformation efficiency percentage (based on PCR confirmation of *hptII* transgene integration) from three consecutive experiments is currently achieving 20.44% for the tossa jute (*C. olitorius*) cultivar JRO524 and 15.55% for the white jute (*C. capsularis*) cultivar JRC212.

## Quantification and statistical analysis

Determine the transformation efficiency using the following formula:

TransformationEfficiency(%)=(NumberofPCRPositivePlants/TotalNumberofImbibedSeeds)×100

Calculate the mean transformation efficiency percentages using Microsoft Excel Worksheet, taking into account data from three consecutive experiments.

**Table:** Transformation efficiency

Experiment Sl. No.	Variety	No of imbibed seed used	PCR positive plants for *hptII* gene	Transformation efficiency (%)	Mean transformation efficiency (%)
1	JRO524	150	30	20	20.44% ± 1.17%
2	JRO524	150	28	18.66	
3	JRO524	150	34	22.66	
1	JRC212	150	22	14.66	15.55% ± 0.58%
2	JRC212	150	23	15.33	
3	JRC212	150	25	16.66	

## Limitations

This protocol for *Agrobacterium*-mediated transformation has been successfully optimized for dicotyledonous bast fiber-producing plants such as jute and flax. However, its applicability to monocotyledonous plants like rice (*Oryza sativa*), maize (*Zea mays*), and wheat (*Triticum aestivum*) has not yet been tested. Further research is needed to determine its effectiveness in monocot and other dicot plants. The application of this seed-piercing method to plants with tiny-sized seeds like mustard (*Brassica* sp.), and poppy (*Papaver somniferum*) can be technically challenging.

## Troubleshooting

### Problem 1

Fungal contamination during co-cultivation incubation of imbibed seeds (step 16).

### Potential solution

• Increase the amount of Bavistin (carbendazim 50%) or consider using alternative fungicides, especially for tossa jute (*C. olitorius*) varieties.
• If fungal contamination persists, try using seeds from a different freshly harvested lot for transformation.

### Problem 2

Co-Cultivation Media dries out during the incubation period (step 39).

### Potential solution

• Optimize the ‘Co-Cultivation Media’ volume; consider increasing it from 3 mL to 5 mL in a 100 mm petri plate.
• Place filter paper on a 1% agar plate to maintain moisture during the co-cultivation period.

### Problem 3

Plants die shortly after being transferred to soil in the greenhouse (step 52).

### Potential solution

• Introduce a hardening step before transferring plants into the soil in the greenhouse. Keep them in a soilrite-mix in the culture room for 7–10 days before the final transfer.
• Alternatively, grow plants in sand for a few weeks before transferring them to soil.

### Problem 4

Sometimes piercing marks (holes) prevail initially in the cotyledon leaf and first true leaf (step 53).

### Potential solution

• There is no need to be concerned about the piercing marks in the cotyledon leaf and first true leaf, as these marks (holes) tend to naturally fill in as the plant develops.

### Problem 5

Occurrence of a high number of PCR-negative plants (step 67).

### Potential solution

• Increase the concentration of hygromycin-B in the selection media.
• Ensure the hygromycin-B stock does not expire and is stored at the appropriate temperature.
• Implement at least three rounds of hygromycin-B selection cycles.

## Resource availability

### Lead contact

Further information and requests for resources and reagents should be directed to and will be fulfilled by the lead contact, Dr. Shuvobrata Majumder (shuvo@ils.res.in).

### Technical contact

Technical questions on executing this protocol should be directed to and will be answered by the technical contact, Dr. Shuvobrata Majumder (shuvo@ils.res.in).

### Materials availability

The current study aims to optimize a protocol for jute as a proof of concept. While no transformed plant has been deposited yet in a public repository, related information is available from the authors upon request.

### Data and code availability

This study does not generate/analyze.

## Supplemental information

Data S1. Complete DNA sequence of pCAMBIA1301, related to step 53
